# Improving the visual quality and delivery of visuospatial and executive function tests on an interactive web and mobile platform

**DOI:** 10.1002/alz70858_107644

**Published:** 2025-12-26

**Authors:** Arturo Del Bosque‐Díaz De León, Mireya Saraí García‐Vázquez, Alejandro Álvaro Ramírez‐Acosta, Sara Aguilar‐Navarro, Alberto Mimenza

**Affiliations:** ^1^ Instituto Politécnico Nacional, Zacatecas, ZT, Mexico; ^2^ Instituto Politécnico Nacional, CITEDI, Tijuana, BJ, Mexico; ^3^ MIRAL R&D&I Multimedia, San Diego, CA, USA; ^4^ Instituto Nacional de Ciencias Médicas y Nutrición “Salvador Zubiran”, Mexico City, Mexico; ^5^ Insituto Nacional de Ciencias Médicas y Nutrición Salvador Zubiran, Mexico, DF, Mexico

## Abstract

**Background:**

One of the tests to evaluate cognitive decline is the Montreal Cognitive Assessment (MoCA), as a result, various technological developments have automated and digitized this test. Some of these systems present the test only in its digitalized form, while others integrate virtual reality and artificial intelligence with sophisticated signal processing methods that take into account the information from each item on the test to obtain systems that support diagnosis for professionals in this field.

This type of system is contributing to the counterbalance of the expected scenario for the year 2050, according to the World Health Organization, which predicts that 140 million people will suffer from dementia.

**Method:**

In this research, we present an automatic method based on image quality to obtain the illustrations made by patients during the visuospatial and executive function tests of the Montreal Cognitive Assessment (MoCA). Specifically, this method performs an automatic analysis and image processing that identifies the illustration with the best focus and quality for each of the three tests: Trail Making, Cube, and Clock.

**Result:**

This optimization in the acquisition of information in cognitive domains, both in visuoconstructional skills and executive functions, provides robustness to our interactive web platform. In addition, when performing the MoCA test, the patient can save an estimated time between 50% and 70% by being able to capture and save the images immediately.

This method avoids the most common problems when capturing images, such as having low‐quality images due to being captured in environments where with too low or too bright lighting, as well as instability in the movement of capture device.

**Conclusion:**

For the test database, the automatic scoring of each MoCA item and the score set for each of the three illustrations by the specialist, the results of our web application are obtained with 100% accuracy and equal to the evaluations carried out by specialists.

The Web platform stores the tests performed so that the specialist doctor can analyze them and continuously monitor patients. This information is managed by the doctor and can be consulted by the patient's caregiver or family member.

